# Outcome Analysis of Breakthrough Invasive Aspergillosis on Anti-Mold Azole Prophylaxis and Treatment: 30-Year Experience in Hematologic Malignancy Patients

**DOI:** 10.3390/jof11020160

**Published:** 2025-02-19

**Authors:** Hiba Dagher, Anne-Marie Chaftari, Andrea Haddad, Ying Jiang, Jishna Shrestha, Robin Sherchan, Peter Lamie, Jennifer Makhoul, Patrick Chaftari, Ray Hachem, Issam Raad

**Affiliations:** Department of Infectious Diseases, Infection Control and Employee Health, The University of Texas MD Anderson Cancer Center, Houston, TX 77030, USA; hrdagher@mdanderson.org (H.D.); andreahaddad95@gmail.com (A.H.); yijiang@mdanderson.org (Y.J.); jshrestha@mdanderson.org (J.S.); rsherchan@mdanderson.org (R.S.); pmlamie@mdanderson.org (P.L.); pchaftari@mdanderson.org (P.C.); rhachem@mdanderson.org (R.H.); iraad@mdanderson.org (I.R.)

**Keywords:** Aspergillosis, *Aspergillus*, invasive Aspergillosis, azole, amphotericin B, prophylaxis, breakthrough fungal infections, invasive fungal infection, anti-mold, antifungal

## Abstract

Background: Anti-mold azoles have improved the outcomes of invasive aspergillosis (IA) when used therapeutically, but they are extensively used as prophylaxis. There are limited data regarding the outcomes of patients with hematologic malignancy who develop breakthrough IA on anti-mold azoles. We aimed to determine whether breakthrough IA on azole prophylaxis shows worse outcomes compared to no prophylaxis. Methods: We compared outcomes including therapy response and mortality between antifungal regimens in hematologic malignancy patients with IA between July 1993 and July 2023. Results: Compared to an amphotericin B-containing regimen (AMB), an anti-mold azole as the primary therapy was independently associated with successful response at the end of therapy (OR = 4.38, *p* < 0.0001), protective against 42-day IA-associated mortality (OR = 0.51, *p* = 0.024) or all cause mortality (OR = 0.35, *p* < 0.0001), and protective against 84-day mortality, both IA-associated (OR = 0.50, *p* = 0.01) and all-cause mortality (OR = 0.27, *p* < 0.0001). Azole prophylaxis was independently associated with higher IA-associated mortality at 42 days (OR = 1.91, *p* = 0.012) and 84 days (OR = 2.03, *p* = 0.004), compared to fluconazole or no prophylaxis. Conclusions: Patients with breakthrough IA on anti-mold azole prophylaxis show a worse prognosis than those on other or no prophylaxis, possibly related to the emergence of azole resistance due to their widespread use as prophylaxis agents. On the other hand, anti-mold azole primary therapy is superior to AMB therapy in the treatment of IA.

## 1. Introduction

Invasive aspergillosis (IA) is a significant cause of morbidity and mortality in immunocompromised patients with hematologic malignancy, particularly those undergoing intensive chemotherapy or hematopoietic stem cell transplant [1,2,3,4]. The incidence of IA is notably high in these populations due to their compromised immune systems, with prevalence rates of up to 10% in high-risk groups such as patients with acute myeloid leukemia or myelodysplastic syndromes undergoing chemotherapy [5,6]. The introduction of azole antifungals in the 1990s marked a turning point in the management of IA. Itraconazole, approved in 1992, was one of the first azoles used for prophylaxis in immunocompromised patients, but it was later replaced by voriconazole in 2002, which became the preferred treatment for IA due to its superior efficacy and tolerability [2,7]. In 2007, posaconazole was introduced and demonstrated further improvement in prophylaxis, especially for preventing IA in neutropenic patients [8].

The Infectious Diseases Society of America (IDSA) issued updated guidelines in 2016 recommending azoles as the primary prophylactic agents for IA in high-risk cancer patients, based on evidence from pivotal clinical trials [1,5,6]. Despite effective prophylaxis, breakthrough IA infections can occur, often due to drug resistance or inadequate therapeutic drug levels. The IDSA guidelines emphasize the importance of therapeutic drug monitoring to optimize antifungal levels and suggest switching to alternative agents such as liposomal amphotericin B (AMB) or echinocandins in cases of breakthrough infection due to factors such as azole resistance and suboptimal therapeutic levels [1,2]. However, there is still limited research regarding the outcomes of patients with hematologic malignancy, and patients who develop breakthrough IA while on anti-mold azole prophylaxis and switch to AMB and/or echinocandin primary therapy, versus those who receive de novo azole primary therapy. In this study, we aimed to compare clinical outcomes between patients with IA on anti-mold azole prophylaxis versus echinocandin vs. non-anti-mold prophylaxis across 30 years of data. We also aimed to compare outcomes based on primary AMB-based therapy and anti-mold azole therapy.

## 2. Methods

### 2.1. Patient Population

This was a retrospective cohort study conducted at the University of Texas MD Anderson Cancer Center (Houston, Texas), encompassing data between July 1993 and July 2023. We included all patients with hematologic malignancy who had documented proven or probable IA during the study period. Baseline characteristics, antifungal therapies used as prophylaxis and primary therapy for IA, and clinical outcomes in these patients were extracted from the hospital’s electronic medical record system. Patient data were stored in password-protected electronic case report forms (REDCap). This study was approved by the institutional review board at MD Anderson. Due to the retrospective nature of the study, informed consent was waived. All patient data were de-identified.

Proven and probable IA were defined according to the European Organization for Research and Treatment of Cancer and Mycoses Study Group criteria EORTC/MSG [9]. Proven IA included documented histopathologic and microbiologic evidence of *Aspergillus* species infection on biopsy tissue samples or culture samples from a normally sterile site. Probable IA was characterized by the presence of radiologic and microbiologic features supporting an IA diagnosis: the presence of nodules or cavities, or a halo or air-crescent sign on chest X-ray or computerized tomography, and documentation of *Aspergillus* infection by direct microscopy or culture in a neutropenic patient with an absolute neutrophil count of 500 cells/µL, who had undergone prolonged steroid therapy, or who had used cytotoxic agents [9,10].

Patients were classified into 3 groups according to antifungal prophylaxis regimen: anti-mold azole-containing prophylaxis (including voriconazole, posaconazole, isavuconazole, itraconazole; group 1 [G1]), echinocandin-containing prophylaxis without azoles (including caspofungin, micafungin, anidulafungin; group 2 [G2]), and no mold prophylaxis or fluconazole-only prophylaxis, since it does not cover mold (group 3 [G3]). Therefore, G1 and G2 represent breakthrough IA infections while on anti-mold prophylaxis. All the patients from these three groups were classified according to primary therapy regimens as well: an AMB-based regimen alone, an azole-containing regimen alone, neither azole nor AMB, or both azole and AMB. AMB includes liposomal AMB or AMB lipid complex. Antifungal prophylaxis was defined as an antifungal agent administered for at least 7 days immediately before an IA diagnosis. Primary antifungal therapy was defined as an antifungal regimen (single-agent or combination) administered for at least 7 consecutive days immediately after IA diagnosis.

### 2.2. Outcomes

Clinical outcomes were compared between different prophylaxis and treatment groups. The primary outcome was treatment success, defined as complete or partial resolution of clinical and radiological signs of infection by the end of antifungal therapy. A favorable response was defined as a complete or partial clinical, radiologic, or microbiologic resolution of IA. Failure to respond was characterized as worsening or stable clinical or radiologic parameters compared with the baseline.

Secondary outcomes included 42-day and 84-day overall and IA-associated mortality, intensive care unit (ICU) admission, and the need for mechanical ventilation. Overall mortality included all deaths that had occurred within 12 weeks after the initiation of antifungal therapy. IA-attributable death was defined as death in a patient without improvement in IA infection within 12 weeks of follow-up. Mortality was attributed to IA if it occurred within 12 weeks of a documented fungal infection without an alternative cause of death.

## 3. Statistical Analysis

Categorical variables were compared between groups using chi-square or Fisher’s exact tests, as appropriate. Continuous variables were compared using the Kruskal–Wallis test for 3-group comparisons and the Wilcoxon rank-sum test for 2-group comparisons. If a significant difference was detected for a variable in a 3-group comparison, then pairwise comparisons were performed, and the significant *p*-values were adjusted using the Bonferroni procedure to control overall type 1 errors in multiple comparisons. Multivariate logistic regression analysis was used to identify the independent predictors of the outcomes, including treatment success and 42-day and 84-day IA-associated and all-cause mortality rates. The logistic regression analyses included all patients except those with primary therapy containing both AMB and azole, to allow for a comparison of the effects of azole and AMB. All tests were 2-sided, with a significance level of 0.05. The statistical analyses were performed using SAS version 9.4 (SAS Institute Inc., Cary, NC, USA).

## 4. Results

A total of 614 patients were compared across the three anti-mold prophylaxis groups: anti-mold azole-containing prophylaxis (G1, n = 163), echinocandin-containing prophylaxis without azoles (G2, n = 62), and no prophylaxis or fluconazole-only prophylaxis (G3, n = 389) (Table 1). Most baseline characteristics were similar between the 3 groups. The median age did vary significantly between groups (*p* < 0.0001), with G3 patients being older. The incidence of invasive pulmonary infections, the most common type of IA, was highest in G2 (90% in G2 vs. 77% in G1, *p* = 0.019). Disseminated infections were more common in G1 (12%) compared to G2 (2%) and G3 (6%) (*p* = 0.005). Leukemia was more common in G1 (77%) and G2 (74%) compared to G3 (61%, *p* < 0.001). Graft versus host disease occurred more frequently in G1 (28%) and G2 (30%) compared to G3 (15%, *p* < 0.001). Additionally, G2 exhibited a significantly higher rate of ICU admissions (*p* < 0.01) and mechanical ventilation (*p* < 0.01).

The 42-day all-cause mortality and IA-associated mortality were significantly higher in G1 compared to G3 (*p* = 0.032 and *p* = 0.02, respectively) (Table 1). Upon multivariate analysis, compared to AMB-containing primary therapy, azole primary therapy was significantly associated with a successful response at the end of therapy (OR = 4.38, *p* < 0.0001) (Table 2). Factors associated with a worse response included the presence of neutropenia at IA onset (OR = 0.45, *p* < 0.001) (Table 2) and the presence of graft-versus-host disease (OR = 0.51, *p* = 0.012), as expected.

On multivariate analysis, azole prophylaxis was independently associated with improved IA-associated 42-day mortality (OR = 1.91, *p* = 0.012) (Table 3) and IA-associated 84-day mortality (OR = 2.03, *p* = 0.004) in patients with breakthrough IA (Table 4) compared to no prophylaxis or fluconazole prophylaxis. Compared to AMB primary therapy, azole primary therapy was protective against IA-associated 42-day mortality (OR = 0.51, *p* = 0.015) (Table 3), all-cause 42-day mortality (OR = 0.35, *p* < 0.0001) (Table 5), IA-associated 84-day mortality (OR = 0.50, *p* = 0.01) (Table 4), and all-cause 84-day mortality (OR = 0.27, *p* < 0.0001) (Table 6).

## 5. Discussion

Our results consistently show that anti-mold azole primary therapy was associated with significantly better clinical outcomes, including therapy response compared to AMB-based primary therapy among patients with hematologic malignancies. Primary therapies with anti-mold azoles were also independently associated with fewer IA-attributable deaths and overall mortality within 6 and 12 weeks after treatment initiation (*p*-values < 0.01) compared to AMB primary therapy. Interestingly, our results show worse outcomes among breakthrough IA patients who were receiving anti-mold azole prophylaxis (*p* = 0.01 for 42-day and *p* = 0.004 for 84-day mortality) compared to patients with fluconazole or no prophylaxis.

The 2016 IDSA guidelines provide comprehensive recommendations for prophylaxis and the treatment of IA. In high-risk patients, such as those undergoing hematopoietic stem cell transplant or receiving intensive chemotherapy, the guidelines recommend posaconazole for prophylaxis. Voriconazole and isavuconazole are also alternatives in high-risk settings [1]. These recommendations are based on several studies that showed the efficacy of azoles as prophylaxis agents. Cornely et al. showed that posaconazole prophylaxis demonstrated superiority over fluconazole and itraconazole prophylaxis in reducing the incidence of IA and improving overall survival in neutropenic patients with acute myeloid leukemia and myelodysplastic syndromes [5]. Wingard et al. demonstrated that voriconazole prophylaxis had comparable efficacy to fluconazole prophylaxis but was better tolerated in allogeneic hematopoietic stem cell transplant recipients [6]. More recently, Walsh et al. compared posaconazole and voriconazole and found that both agents were equally effective in preventing IA, though posaconazole had a more favorable safety profile [11]. However, when breakthrough infections on prophylaxis or azole resistance occur in high-risk patients, the IDSA guidelines recommend switching to alternative agents like liposomal AMB or echinocandins for primary treatment, although these options come with limitations, such as higher toxicity and reduced efficacy, compared to azoles [1].

AMB-based treatments have long been employed for IA across various patient groups. However, several studies have shown that in hematologic malignancy patients with IA, AMB with newer lipid formulations did not significantly improve response rates [12,13] and was linked to worse clinical outcomes when used as a single-agent therapy [10,14]. On the other hand, several studies have shown the efficacy of azoles in the primary and salvage treatment of IA [2,15,16,17,18]. In 2002, Herbrecht et al. concluded that voriconazole was more effective and had a better safety profile than AMB for the primary treatment of IA [2]. Upton et al. reported a lower IA-related mortality in hematopoietic stem cell transplant recipients treated with voriconazole [18]. In addition, Maertens et al. showed that posaconazole was non-inferior to voriconazole and was a safer alternative for the primary treatment of IA [15].

With the widespread use of azoles as prophylaxis therapy and the recommendations to switch to AMB regimens for breakthrough IA infections, our results raise the following question: Could the overuse of azoles as anti-mold prophylaxis be hindering their use as effective primary therapy options in patients with hematologic malignancies and in stem cell transplant recipients and contributing to an increase in *Aspergillus* species resistant to azoles?

The emerging prevalence of azole-resistant *Aspergillus* presents a significant global challenge, particularly for immunocompromised patients at risk of IA. Several studies have documented the emergence of triazole resistance and cross-resistance among *Aspergillus* species isolates [19,20]. Studies have consistently shown resistance rates between 3% and 10%, with certain regions such as Europe and Asia having a higher prevalence rate compared to the United States [21,22,23]. Lockhart et al. highlighted the emerging threat of azole resistance in the United States, noting that although resistance rates are lower than in Europe, there is growing concern due to the increasing use of antifungal agents in both clinical and agricultural settings [23].

The emergence of these resistant strains has severe clinical consequences, as mortality rates in patients with azole-resistant IA can reach as high as 88%, compared to 30–50% in azole-susceptible infections [21,24]. This has led to a growing reliance on alternative treatments, such as liposomal AMB and echinocandins, although these options are often less effective, as shown in the results of this present study, compared to azoles and may be associated with higher toxicity [1,2].

Despite growing resistance, azoles remain the first-line therapy for IA, but alternative therapies and combination treatment may become more necessary as resistance patterns evolve. The overuse of azoles as IA prophylaxis in immunocompromised patients may be contributing to the rise in resistance and may lead to worse clinical outcomes in immunocompromised patients with breakthrough IA. These patients are often switched to AMB-based anti-mold regimens that are less effective as primary therapy compared to azoles, as shown by our results. In a randomized trial, Marr et al. showed the efficacy of voriconazole as a primary therapy in the treatment of IA while also demonstrating lower mortality rates at 6 weeks in a subgroup of patients with high galactomannan levels treated with a combination of anidulafungin and voriconazole compared to voriconazole monotherapy [16]. This suggests the possible efficacy of switching to a different azole and the addition of an echinocandin as a better primary treatment option. Future studies should evaluate azole resistance in patients who end up with breakthrough IA and what would be the optimal therapeutic option while considering the possibility of combination therapy.

The primary limitations of this study stem from its retrospective design and the inherent variability associated with its 30-year cohort. Over this period, significant changes have occurred, including updates to IDSA guidelines, evolving therapeutic practices, and advancements in antifungal treatment options. In addition, there have been improvements in cancer management and chemotherapy regimens, the specifics of which were not captured for all patients in this cohort. To address these limitations, we accounted for the three decades in our multivariate analysis.

## 6. Conclusions

Patients who develop breakthrough IA while receiving anti-mold azole prophylaxis show a worse prognosis than those receiving fluconazole or no prophylaxis, possibly due to the emergence of azole resistance with the wide use of anti-mold azole prophylaxis. Moreover, anti-mold azole primary therapy is superior to AMB therapy in the treatment of IA. Further studies are needed to assess switching to a combination of a different azole with an echinocandin as primary therapy, as well as to evaluate emerging azole resistance in this patient population.

## Figures and Tables

**Table 1:** Comparison of patients with different prophylaxis regimens.

Variables	Anti-Mold Azole-Containing Prophylaxis	Echinocandin-Containing Prophylaxis (Without Azoles)	No Prophylaxis or Prophylaxis with Fluconazole Only	*p*-Value	Pairwise Comparisons with Significant Differences *
	(G1)	(G2)	(G3)		
	(N = 163)	(N = 62)	(N = 389)		
IA diagnosis period, n (%)				0.17	
1993–2003	63 (39)	13 (21)	136 (35)		
2004–2013	48 (29)	23 (37)	116 (30)		
2014–2023	52 (32)	26 (42)	137 (35)		
Age (years), median (IQR)	53 (39–63)	54 (39–68)	59 (46–68)	<0.0001	G1 vs. G3: *p* < 0.001
Sex, male, n (%)	107 (66)	32 (52)	231 (59)	0.13	
Diagnosis of IA, n (%)				0.07	
Definite IA	56/161 (35)	14/61 (23)	99/384 (26)		
Probable IA	105/161 (65)	47/61 (77)	285/384 (74)		
Type of IA, n (%)					
Invasive pulmonary infection	126 (77)	56 (90)	333 (86)	0.019	None
Disseminated infection	20 (12)	1 (2)	22 (6)	0.005	G1 vs. G2: *p* = 0.042; G1 vs. G3: *p* = 0.023
Localized infection	5 (3)	2 (3)	24 (6)	0.25	
Sinus infection	14 (9)	5 (8)	13 (3)	0.023	G1 vs. G3: *p* = 0.027
Type of hematologic malignancy, n (%)					
Leukemia	126 (77)	46 (74)	237/388 (61)	<0.001	G1 vs. G3: *p* < 0.001
Lymphoma	32 (20)	11 (18)	95/388 (24)	0.29	
Myeloma	2 (1)	2 (3)	36/388 (9)	0.001	G1 vs. G3: *p* = 0.002
Transplant within 1 year before IA, n (%)	72/162 (44)	26 (42)	115/384 (30)	0.003	G1 vs. G3: *p* = 0.003
Type of transplant within 1 year before IA, n (%)				<0.0001	
Autologous	3/69 (4)	1/24 (4)	33/113 (29)		G1 vs. G3: *p* < 0.001
Allogeneic	66/69 (96)	23/24 (96)	80/113 (71)		G2 vs. G3: *p* = 0.03
GVHD, n (%)	46/162 (28)	18/61 (30)	57/383 (15)	<0.001	G1 vs. G3: *p* < 0.001; G2 vs. G3: *p* = 0.014
Neutropenia (ANC < 500 cells/µL) at IA onset, n (%)	79/161 (49)	29/61 (48)	154/372 (41)	0.22	
*Aspergillus* species				0.85	
*Asp. terreus* (alone or mixed)	28/150 (19)	13/61 (21)	74/357 (21)		
Other *Asp*. species	122/150 (81)	48/61 (79)	283/357 (79)		
Primary antifungal therapy, n (%)					
AMB (but no azole)	48 (29)	17 (27)	122 (31)	0.78	
Containing azole (but no AMB)	48 (29)	16 (26)	177 (46)	<0.001	G1 vs. G3: *p* = 0.002; G2 vs. G3: *p* = 0.007
No AMB and no azole	11 (7)	14 (23)	39 (10)	0.002	G1 vs. G2: *p* = 0.002; G2 vs. G3: *p* = 0.009
Containing both AMB and azole	56 (34)	15 (24)	51 (13)	<0.0001	G1 vs. G3: *p* < 0.001; G2 vs. G3: *p* = 0.044
ICU stay, n (%)	56/162 (35)	36 (58)	140/388 (36)	0.003	G1 vs. G2: *p* = 0.004; G2 vs. G3: *p* = 0.003
Mechanical ventilation, n (%)	42/162 (26)	30 (48)	104/387 (27)	0.002	G1 vs. G2: *p* = 0.004; G2 vs. G3: *p* = 0.002
Final response to therapy, n (%)				0.008	
Success	53/156 (34)	22/59 (37)	181/378 (48)		G1 vs. G3: *p* = 0.013
Failure	103/156 (66)	37/59 (63)	197/378 (52)		
42-day mortality after IA diagnosis, n (%)	73/163 (45)	35 (40)	129/388 (33)	0.032	G1 vs. G3: *p* = 0.03
IA-associated 42-day mortality after IA diagnosis, n (%)	55/157 (35)	19/61 (31)	88/374 (24)	0.02	G1 vs. G3: *p* = 0.019
84-day mortality after IA diagnosis, n (%)	86/162 (53)	30 (48)	170/388 (44)	0.13	
IA-associated 84-day mortality after IA diagnosis, n (%)	63/153 (41)	22/60 (37)	110/366 (30)	0.044	G1 vs. G3: *p* = 0.042

IA = invasive aspergillosis; IQR = interquartile range; ANC = absolute neutrophil count. * The *p*-values for significant differences have been adjusted by Bonferroni procedure to control overall type 1 error for multiple comparisons.

**Table 2:** Multivariate logistic regression model of independent predictors of successful response to IA therapy.

Independent Predictors	aOR	95% CI	*p*-Value
Type of hematologic malignancy			0.033
Myeloma	2.67	1.09–6.58	
Leukemia or lymphoma	Reference		
Neutropenia at IA onset	0.45	0.30–0.69	<0.001
Graft-versus-host disease	0.51	0.30–0.86	0.012
Primary therapy containing *			<0.0001
Amphotericin B	Reference		
Azole	4.38	2.82–6.81	<0.0001
Neither	1.25	0.62–2.50	0.53

aOR = adjusted odds ratio; 95% CI = 95% confidence interval. * For direct comparison of primary therapies containing azole vs. AMB, patients with primary therapy containing both AMB and azole were excluded from analysis.

**Table 3:** Multivariate logistic regression model of independent predictors of IA-associated 42-day mortality.

Independent Predictors	aOR	95% CI	*p*-Value
Time period of IA diagnosis			0.01
1993–2003	Reference		
2004–2013	0.69	0.40–1.20	0.19
2014–2023	0.36	0.19–0.70	0.002
BMT within 1 year	0.60	0.37–0.98	0.04
Neutropenia at IA onset	1.59	1.02–2.49	0.043
Prophylaxis			0.042
Anti-mold azole-containing agent(s)	1.91	1.15–3.15	0.012
Echinocandin-containing agent(s)	1.23	0.58–2.58	0.59
No prophylaxis or fluconazole only	Reference		
Primary therapy containing *			<0.001
Amphotericin B	Reference		
Azole	0.51	0.30–0.88	0.015
None of above	1.75	0.87–3.50	0.12

* For direct comparison of primary therapies containing azole vs. AMB, patients with primary therapy containing both AMB and azole were excluded from analysis.

**Table 4:** Multivariate logistic regression model of independent predictors of IA-associated 84-day mortality.

Independent Predictors	aOR	95% CI	*p*-Value
Time period of IA diagnosis			0.008
1993–2003	Reference		
2004–2013	0.67	0.39–1.14	0.14
2014–2023	0.37	0.20–0.69	0.002
Neutropenia at IA onset	1.68	1.10–2.55	0.017
Prophylaxis			0.017
Anti-mold azole-containing agent(s)	2.03	1.25–3.30	0.004
Echinocandin-containing agent(s)	1.26	0.62–2.58	0.52
No prophylaxis or fluconazole only	Reference		
Primary therapy containing *			0.004
Amphotericin B	Reference		
Azole	0.50	0.30–0.85	0.01
None of above	1.34	0.67–2.65	0.41

* For direct comparison of primary therapies containing azole vs. AMB, patients with primary therapy containing both AMB and azole were excluded from analysis.

**Table 5:** Multivariate logistic regression model of independent predictors of all-cause 42-day mortality.

Independent Predictors	aOR	95% CI	*p*-Value
Neutropenia at IA onset	2.25	1.52–3.34	<0.0001
Primary therapy containing *			<0.0001
Amphotericin B	Reference		
Azole	0.35	0.23–0.53	<0.0001
None of above	1.30	0.72–2.33	0.38

* For direct comparison of primary therapies containing AMB vs. azole, patients with primary therapy containing both AMB and azole were excluded from analysis.

**Table 6:** Multivariate logistic regression model of independent predictors of all-cause 84-day mortality.

Independent Predictors	aOR	95% CI	*p*-Value
Invasive pulmonary infection	1.91	1.10–3.34	0.023
Neutropenia at IA onset	2.43	1.63–3.60	<0.0001
Primary therapy containing *			<0.0001
Amphotericin B	Reference		
Azole	0.27	0.17–0.41	<0.0001
None of above	0.95	0.52–1.73	0.86

* For direct comparison of primary therapies containing azole vs. AMB, patients with primary therapy containing both AMB and azole were excluded from analysis.

## Data Availability

The original contributions presented in this study are included in the article. Further inquiries can be directed to the corresponding author.
